# Effect of different topical fluoride applications on the surface roughness of a colored compomer

**DOI:** 10.1590/S1678-77572010000200012

**Published:** 2010

**Authors:** Aysun AVŞAR, Nuray TULOGLU

**Affiliations:** 1 DDS, PhD, Assistant Professor, Department of Pediatric Dentistry, Faculty of Dentistry, Ondokuz Mayis University, Samsun, Turkey.; 2 DDS, Graduate Student, Department of Pediatric Dentistry, Faculty of Dentistry, Ondokuz Mayis University, Samsun, Turkey.

**Keywords:** Colored compomer, Conventional compomer, Resin-modified glass-ionomer, Acidulated phosphate fluoride, Neutral sodium fluoride, Surface roughness

## Abstract

**Objective:**

The aim of this study was to evaluate the effect of neutral sodium fluoride (NNaF) gel and acidulated phosphate fluoride (APF) gel on the surface roughness of colored compomer (Twinky Star), conventional compomer (Compoglass F) and resin-modified glassionomer cement (RMGIC) (Photac-Fil).

**Material and Methods:**

A total of 45 standardized disc-shaped specimens were prepared for each material. After 24 h, finishing and polishing of specimens were done with aluminum oxide disc. Surface treatments with topical fluoride agents or distilled water (control) were performed four times, and interspersed with 8 pH cycles, simulating high cariogenic challenges. After the treatment, the surface roughness (Ra) was determined using a profilometer. In each group, specimens with Ra closest to the mean were examined with a scanning electron microscope (SEM) at ×1,000 and ×3,500 magnifications. Two-way ANOVA was used to evaluate Ra measurements, and the differences in Ra values between subgroups for each material and each topical applications were compared by Tukey’s highly significant difference pairwise comparisons.

**Results:**

No statistically significant difference in Ra between the Twinky Star and Compoglass F was found. However, Photac Fil showed significantly higher Ra than these materials after all surface treatments. There was a general trend of Ra increase from controls to NNaF and APF gels for all materials. SEM observations revealed that the surface micromorphology of Twinky-Star did not differ significantly from that of Compoglass F.

**Conclusion:**

Both the compomers and the RMGIC showed significantly higher surface roughness when subjected to APF gel application.

## INTRODUCTION

Polyacid-modified resin composites, commonly known as "compomers" are a group of esthetic materials for anterior and posterior restorations of primary teeth^14,18^. They were introduced in the early 1990s with claims that they combined the mechanical and esthetic properties of composites with the fluoride-releasing advantages of conventional glass-ionomer cements (GICs)^17^.

Colored compomers, which can be produced by adding a small amount of glitter particles (mainly silicates from kali) to conventional compomers, producing materials with pink, green, blue, silver, orange, lemon or gold shades, have been available for the restoration of primary molars for over 4 years^4,14,17^. When they are asked for a choice, some children prefer tooth-colored, imperceptible dental restorations, while other children enjoy a colorful filling material for their primary teeth^11^. Even though colored compomer is made to be decorative, it has physical properties that apparently are sufficient to hold up in the mouth until the restored primary tooth is lost^5^. In a study of Croll, et al.^5^, a second primary molar restored in a 8-year-old girl with a colored compomer, and reported that the restoration was intact and serving its purpose well 10 months after placement.

Previous studies have shown that topical fluoride application to compomers could increase the surface roughness of this materials^3,7,30^-32. The clinical significance of the increased surface of the materials covers the increased plaque adhesion and its harmful effects on the tooth and periodontium, to surface discoloration and fatigue failure^2-3,12,19,22,29^. The amount of plaque correlates with the surface roughness of compomers, and the fluoride-releasing capacities of these materials do not efficiently prevent the attachment and viability of *Streptococcus mutans*^23,26^.

No previous study has addressed the effects of topical fluoride agents on colored compomer in the dental literature. Therefore, the aim of the present study was to evaluate the effects of acidulated phosphate fluoride (APF) gel and neutral sodium fluoride (NNaF) gel treatment on the surface roughness of colored compomer. The tested null hypothesis was that topical fluoride applications have greater influence on the surface roughness of the colored compomer because of the glitter particles added to these materials compared to conventional compomer and resin-modified glass-ionomer (RMGIC).

## MATERIAL AND METHODS

The restorative materials used in this study together with information on their basic composition and particle size are listed in Figure 1.

**Figure 1:** Manufacturers, types and components of the restorative materials used in the study

**Material**	**Type**	**Components**	Particle Size(μm) *
Photac- Fill Aplicap (ESPE GmbH Seefeld, Germany)	Resin- modified glassionomer composite	Na-Ca-Al-La-fluorosilicate glass, polyacrylic acid, maleic acid, HEMA	7-40
Twinky-Star (Voco Cuxhaven, Germany)	Polyacid-modified resin composite	Ba-Al- Str-fluorosilicate glass, Silicon dioxide, BisGMA, UDMA, carboxylic acid modified methacrylate, camphorquinone, BHT	0.4-3.0
Compoglass F (Vivadent Ets Liechtenstein, Germany)	Polyacid-modified resin composite	Ba-Al-fluorosilicate glass, BisGMA, UDMA; TEGDMA; cyclo-aliphatic dicarboxylic acid dimethacrylate	0-2-3.0

* As disclosed by the manufacturers. Abbreviations: Bis-GMA: bisphenol A glycidyl methacrylate; HEMA: 2-hydroxyethyl methacrylate; UDMA: urethane dimethacrylate; TEG-DMA: triethylene glycol dimethacrylate; BHT: butylated hydroxytoluene.

### Specimen Preparation

Sixty disc-shaped specimens (4 mm in diameter x 2 mm in height) of each material were made according to the manufacturers’ instructions. The material was placed into a splitring stainless-steel mould. The surface of the specimens was covered with a Mylar matrix strip that was pressed using microscopic glass slide with a load of 500 g for 30 s to remove the excess material. The specimens were then polymerized according to the recommended exposure times through the polyester strip with a quartztungsten-halogen (QTH) light-curing unit (Astralis 3, Ivoclar Vivadent Inc., Liechtenstein) with an output of 600 mW/cm^2^. The output from the curing light was periodically monitored with a curing radiometer (Model 100; Demetron, Kerr Corp, CA, USA). Following light curing, specimens were removed from the mould and stored in distilled water at 37°C for 24 h. Finishing and polishing were carried out with aluminum oxide disks (Soflex; 3M/ESPE, St. Paul, MN, USA) at medium, fine, and superfine grits while keeping the material surface wet. For every sequence, 10 strokes were made using a low-speed handpiece in one direction. Ultrasonic cleaning of the polished specimens was performed for 2 min in distilled water to remove any surface debris (Eurosonic energy, Euronda SpA, Italy).

### Surface treatment pH-Cycling protocol

Surface treatments consisted of 2%NNaF gel (Sultan Topex neutral pH gel, Sultan Dental Products, USA), 1.23%APF gel (Sultan Topex APF gel), and distilled water applications.

A total of sixty specimens were made for each material, which were further divided into two groups serving as test (n= 45) and control specimens (n=15). Depending on the text group, NNaF gel, APF gel, or 0.08mL distilled water was applied over the specimen’s upper surface for 4 min. The specimens were then rinsed with deionized water, were subjected to a pH-cycling regimen, as proposed by Featherstone, et al.^10^ and modified by Serra and Curry^21^. The samples were immersed in 5 mL of the demineralizing solution (2.0 mM of calcium and 2.0 mM of phosphate in a buffer solution of 74.0 mM of acetate at pH 4.3) for 6 h at 37°C, followed by rinsing with distilled- deionized water and storage in 5 mL of remineralizing solution (1.5 mM of potassium chloride in a buffer solution of 20 mM of hydroxymethyl-aminomethane at pH 7.0) for 18 h at 37°C. This protocol was performed over 2 consecutive days. The specimens remained immersed in 5 mL of remineralizing solution at 37°C for 1 week. The surface treatment of specimens, followed by demineralization and remineralization cycles, was performed during a period of 4 weeks, amounting to 4 surface treatments applications interspersed with eight pH cycles^25^.

### Surface Roughness Measurement

The mean surface roughness values (Ra-µm) for all specimens was measured using a profilometer (Mitutoyo Surf Test 402 Analyzer; Mitutoyo Corp, Tokyo, Japan). To measure the roughness profile value, the diamond stylus was moved across the surface under a constant load of 3.9 mM. The instrument was calibrated using a standard reference specimen, and then set to travel at a speed of 0.1 mm/s with a range of 600 µm during testing. This procedure was repeated 3 times for each specimen and the average value was considered to be the Ra value.

### Scanning Electronic Microscopy (SEM) Analysis

In each group, specimens with Ra closest to the mean were sputter coated with gold (S150B; Edwards, Crawley, England) and examined under a field emission scanning electron microscope (SEM) (JSM-6335F, JEOL, Tokyo, Japan). The SEM micrographs were made at ×1000 and 3,500 magnification for visual inspection.

### Statistical Analysis

The measurements controlled for normality assumptions, they were found to be normally distributed. Therefore, two-way ANOVA was used to evaluate Ra measurements and then the differences in Ra values between subgroups for each material and each topical applications were compared by Tukey’s highly significant difference (HSD) pairwise comparisons. All statistical analyses were performed with the SPSS statistical software package, and all results were evaluated at the 5% significance level.

## RESULTS

### Surface Roughness

Comparisons of the of surface roughness means among and within the restorative materials are listed in Table 1.

**Table 1:** Comparison of surface roughness means (Ra; mean ± SD, micrometers) among and within the restorative materials.

**MATERIAL**	**Initial^1^**	**APF group^2^**	**NNaF group3^3^**	**Distilled water^1^**
Photac-Filª	0.035 ± 0.02	1.281 ± 0.24	0.783 ± 0.05	0.062 ± 0.05
Twinky-Star^b^	0.029 ± 0.01	0.116 ± 0.08	0.042 ± 0.03	0.035 ± 0.02
Compoglass F^b^	0.022 ± 0.02	0.094 ± 0.06	0.031 ± 0.01	0.028 ± 0.01

There was no statistically difference among groups with the same superscript numbers and materials with the same superscript letters (p>0.05). SD= standard deviation

When the surfaces roughness of the groups were compared in terms of the materials, except for the initial groups, significant differences were found between Photac-Fil and Twinky Star (p<0.05), and also between Photac-Fil and Compoglass F for all subgroups (p<0.05). However there was no significance difference between Twinky Star and Compoglass F (p>0.05).

According to the surface treatments, significant differences were found among the subgroups of Photac-Fil (p<0.001). For Twinky Star and Compoglass F, the surface roughness of the APF gel group was statistically higher than that of the NNaF gel group (p<0.001), distilled water subgroup (p<0.001) and control group (p<0.001), which did not differ significantly (p>0.05) from each other.

### SEM Analysis

**Figure 2:**
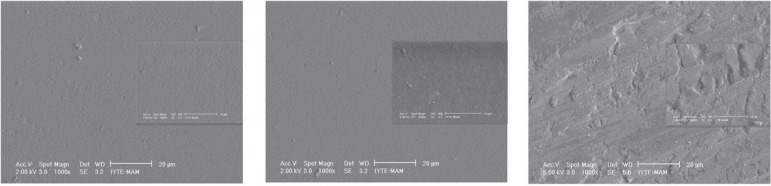
SEM micrographs of the specimens after polishing

**Figure 3:**
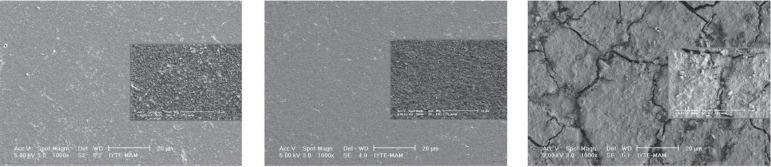
SEM micrographs of the specimens after distilled water application

SEM micrographs of the specimens after polishing are shown in Figure 2. According to the surface treatment protocol, SEM observation showed that the smoothest surface was obtained with the distilled water group of all restorative materials (Figure 3).

**Figure 4:**
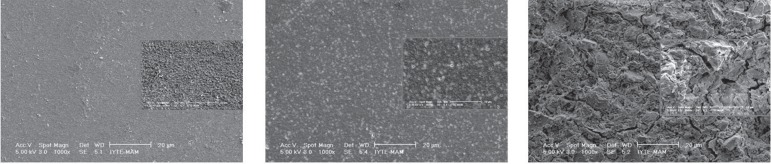
SEM micrographs of the specimens after NNaF gel application

The surface of the NNaF gel group of Twinky Star and Compoglass F appeared smoother with the numerous small voids and porous when compared to the Photac-Fil (Figure 4). For the Photac-Fil, the NNaF gel groups showed significant matrix changes (Figure 4c) when compared to the distilled-water group (Figure 3c).

**Figure 5:**
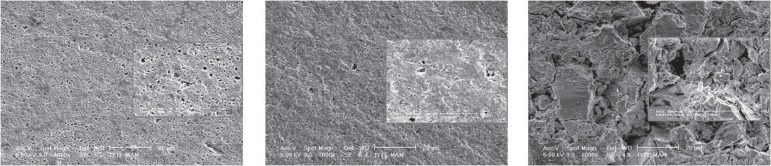
SEM micrographs of the specimens after APF gel application

APF gel application created the roughest surface among the subgroups for all materials. The small voids after NNaF gel application and the later enlarged voids after APF gel application could be readily differentiated from the SEM micrographs for Twinky Star and Compoglass F (Figure 5a and Figure 5b). Photac-Fil showed severe cracks and larger voids (Figure 5c) when compared to the other materials. Numerous cracks noticed in the water group were disappeared after NNaF application.

## DISCUSSION

In the present study, although Twinky Star had visually more surface roughening than Compoglass F, the profilometry data revealed no significant difference between these materials (Table 1). Thus, the null hypothesis was rejected.

There are no previous studies about physical properties of colored compomer in dental literature. In the present *in vitro* study, the surface roughness was analyzed because it has been demonstrated that surface texture can play a role in bacterial colonization of restorative materials^8^. Mean surface roughness of 0.2 µm has been found to be critical for a dramatic increase in the colonization of cariogenic microorganisms, and an extensive review of the literature on this topic has been published^12^. Although contradictory results have been reported in the literature regarding the effect of surface properties on these phenomena^9^, the adherence and metabolic properties of microorganisms in the mouth are well known to be the primary causes of variety of conditions including dental caries and inflammatory diseases of the gingival and periodontal tissues^12^.

The characteristics of filler particles, such as their composition, shape and size, as well as the entanglement of the resin and inorganic matrices, play an important role in the behavior of restorative materials subjected to topical fluoride applications^6,15,16,26^. McCabe^16^ and Meyer, et al.^17^ suggested that the amount of resin matrix increases from RMGIC to polyacid-modified composite resin, and this matrix is obviously not susceptible to degradation by topical fluoride applications. The absence of significant differences among subgroups for Twinky Star and Compoglass F may be attributed to the similar size of their particles (Table 1). On the other hand, for Photac-Fil, whose particle size is larger and amount of resin matrix is lesser, showed very rough surface with voids present confirmed by profilometry.

For Photac-Fil, a significant surface roughness was detected for specimens treated both NNaF gel and APF gel (Table 1), as others have reported^13,15,31^. The filler particles were eroded and partially or completely exposed because of the absence of the surrounding matrix, and the matrix also appeared to be severely degraded (Figure 3c, 4c). According to Turssi, et al.^25^, based on the erratic behavior pattern shown by Photac-Fil as a result of NNaF gel and APF gel treatment, degradation depends not only on the pH of the gel, but probably also on the gel’s ability to form a complex structure with the metal ions of the restorative material. Yip, et al.^30^ suggested that Ra values of the Photac-Fill were comparable to the conventional GICs, and according to these investigators it is possible that stresses built up in the glass particle-resin matrix interfaces. Hadley, et al.^13^ and Billington, et al.^1^ demonstrated that immersion of Photac-Fil in 0.02% NNaF solution for 24 h results in surface roughening of about 310-370%. Strother, et al.^24^, in contrast, reported that a significant surface roughening could not be detected when Photac-Fil was treated once with a NNaF gel for 4 min. This difference may be due to the differences in methodologies used.

Unlike the results observed with Photac-Fill, Twinky Star and Compoglass F showed insignificant surface roughness after NNaF gel application (Figure 2b, Figure 3b) compared to distilled water (Figure 2a, Figure 3a). According to Meyer, et al.^17^, this may be attributed to the different nature of these materials because for compomers, glass particles are partially silanized, providing a direct bond with the resin matrix. In this way, Twinky Star and Compoglass F behave more like resin composite than Photac-Fil, which may explain the fact that the NNaF gel had no effect on these materials.

APF gel contains hydrofluoric acid and phosphoric acid^15^. Hydrofluoric acid is more destructive than phosphoric acid because it can etch glass at lower temperatures and dissolves the composite filler particles resulting in a pitted surface^27^. This might be a possible explanation for the findings of the present study. Compoglass F and Twinky Star had significantly rougher surface after APF gel application compared to NNaF gel and distilled water (Table 1). Similar results were observed in study of Yip, et al.^31^. This roughened surface may contribute to plaque accumulation and may produce surface staining of the materials^12,23^. Controversial finding has been reported on the susceptibility to Compoglass F treated with fluoride gels by Cehreli, et al.^3^. Because of the differences in methodologies used, it is difficult to compare the present observations to those of previous studies.

Further investigations are needed to elucidate the long-term effects of topical fluoride agents on colored compomer under *in vivo* conditions.

## CONCLUSIONS

In the light of the results obtained in the present study, it was observed that: (i) APF gel application increased the surface roughness of Twinky Star, Compoglass F and Photac–Fil restorations; (ii) The surface roughness of Twinky Star and Compoglass F was not significantly affected by application of neutral fluoride gels (p>0.05); (iii) Photac–Fil was significantly affected by applications of any of the fluoride gels.
